# Complete genome sequences of mycobacteriophages Ashwin from cluster O and Chanagan from cluster A1

**DOI:** 10.1128/mra.01028-24

**Published:** 2024-11-14

**Authors:** Edith Erika Machowski, Khel Gordhan, Chandni Madhav, Christopher Ealand, Bavesh Kana

**Affiliations:** 1 School of Pathology, Faculty of Health Sciences, University of the Witwatersrand, National Health Laboratory Service, Johannesburg, South Africa; 2 Biology Department, Illinois Wesleyan University, Bloomington, Illinois, USA; Queens College Department of Biology, Queens, New York, USA

**Keywords:** mycobacteriophage, *Mycobacterium*, bacteriophage therapy, genome

## Abstract

Ashwin and Chanagan are double-stranded *siphoviral* mycobacteriophages isolated from a single soil sample from a lavender flowerpot in Johannesburg, South Africa. Both are capable of infecting and lyzing *Mycobacterium smegmatis* and are predicted to contain 122 and 91 open reading frames, respectively, most without known function.

## ANNOUNCEMENT

Interest in mycobacteriophages for use in clinical applications has led to increased efforts to collect and classify new phages (1–3). Mycobacteriophages Ashwin and Chanagan were isolated from a soil sample in Johannesburg, South Africa (24 September 2022; GPS coordinates −26.177°, 28.283°), from lawns of *Mycobacterium smegmatis* strain mc^2^155 cultured at 37°C on Middlebrook 7H10 agar plates (4). Suspensions filtered through a 0.22 µm membrane in mycobacteriophage buffer were poured in 5-mL top agar overlays after adsorption to mc^2^155. After one round of purification, a single plaque was used to generate high-titer stocks, pooled from multiple mc^2^155 lawns with near-confluent plaques. These were used for transmission electron microscopy and whole genome sequencing. Genomic DNA was extracted using the Wizard Genomic DNA purification kit (Promega) as per the manufacturer’s instructions. Library preparation was performed using the NEBNext Ultra II FS kit, followed by enzymatic fragmentation (200 bp) with AMPure XP beads, end repair, ligation with Illumina-specific adapter sequences, and size selected again. Both genomes were sequenced on Illumina’s NextSeq500 platform. Sequences were trimmed using Illumina Experiment Manager v1.9 before genome assembly, using default settings. A total number of paired-end reads (2 × 150 bp) were 377,899 for Ashwin and 391,550 for Chanagan. Single mycobacteriophage contigs were assembled for each mycobacteriophage and checked for quality, completeness, accuracy, and genomic termini, using Newbler v.2.9 (5) and Consed v.29.0 (6). Genome annotations were refined by comparison to other similar mycobacteriophages using BLASTn alignments NCBI (https://blast.ncbi.nlm.nih.gov/) and Phamerator (7) and protein domain predictions using the DNAMaster annotation suite [v5.23.6, https://phagesdb.org/DNAMaster (8)], GeneMark (v2.5p (9)), and HHpred (10). Aragorn (V2.0) (11) and tRNAscan-SE (V2.0) (12, 13) were used to search for tRNA and tmRNA. All searches were performed using default settings, except for tRNAscan-SE which was adjusted as follows: Sequence source: “Bacterial”; Search mode: “Infernal without HMM”; Extended Options: Check “Disable pseudo gene checking”; Check “Show primary and secondary structure components to scores”; and Genetic Code for tRNA isotype Prediction: “Universal” and a Score cut-off of 17.

**Fig 1:**
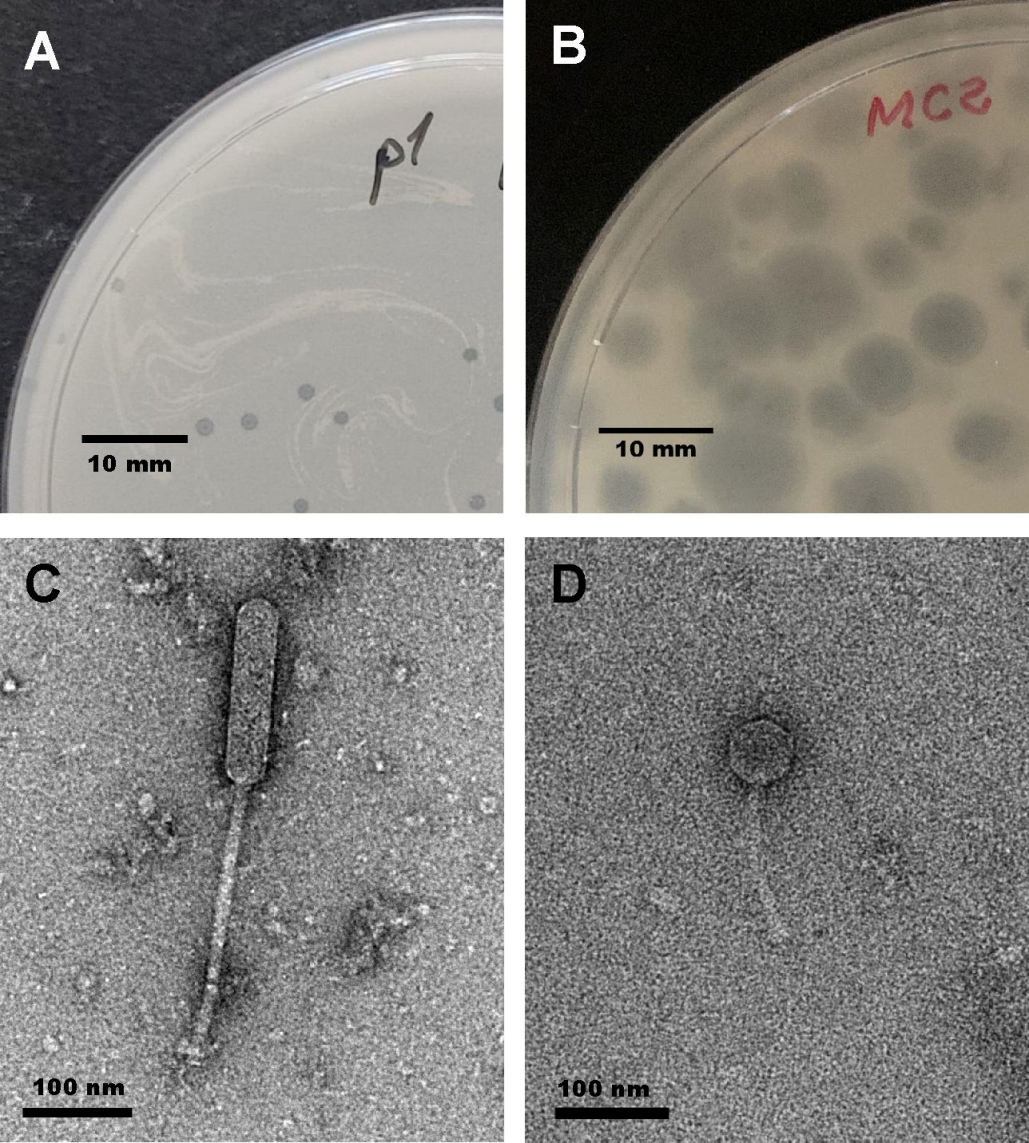
Morphological characterization of mycobacteriophages Ashwin and Chanagan. (**A**) Plaques of mycobacteriophage Ashwin on a lawn of *Mycobacterium smegmatis* are clear and ~1 mm in diameter. (**B**) Plaques of mycobacteriophage Chanagan on a lawn of *Mycobacterium smegmatis* are turbid with a clear center, ~3–5 mm in diameter, which is typical of integrating phages of cluster A1. (**C**) Transmission electron micrograph of an Ashwin virion. The capsule is prolate, with a length of ~169 nm and a width of ~40 nm. The noncontractile tail is ~260 nm long. Siphoviruses with prolate capsules belong to cluster O. (**D**) Transmission electron micrograph of a Chanagan virion with an icosahedral capsule ~60 nm wide and a noncontractile tail with a length of ~142 nm. Sizes are indicated by scale bars at the bottom left of each figure. Images of virions negatively stained with 2% uranyl acetate were captured on a FEI T20 transmission electron microscope at 200kV.

Ashwin produced small, clear plaques on *M. smegmatis* lawns (Fig. 1A), while Chanagan formed larger turbid plaques with clear centers (Fig. 1B). Both phages are *siphoviral* with noncontractile tails. The capsules are prolate for Ashwin (Fig. 1C) and icosahedral for Chanagan (Fig. 1D). Genome analysis assigned Ashwin to cluster O. The 69,731 base pair double-stranded DNA genome with a G+C content of 65.5% bears a GTCT 3′ sticky overhang. Its closest relative is mycobacteriophage Firecracker [99.07% BLASTn at NCBI; GenBank number NC_023712.1]. Of the 122 putative genes encoded, 82 (67.2%) have no known function. Genome analysis assigned Chanagan to cluster A1. Its double-stranded DNA genome, 50,727 base pairs in length, has a G+C content of 63.7%, harbors a CGGATGGTAA 3′ sticky overhang, and is predicted to contain 91 open reading frames. Its closest relative is mycobacteriophage RidgeCB [98.26% BLASTn at NCBI; Genbank number NC_023710.1]. Of the 91 putative genes encoded, 62 (68.1%) have no known function. There are no predicted tRNA or tmRNA in either phage.

## Data Availability

The Ashwin genome sequence is available in GenBank under accession number PP763601. The raw sequence reads are available in the SRA under BioProject accession number PRJNA1102826. The Chanagan genome sequence is available in GenBank under accession number PP763602. The raw sequence reads are available in the SRA under BioProject accession number PRJNA1102824.
